# Supplementary material for: Secreted dengue virus NS1 from infection is predominantly dimeric and in complex with high-density lipoprotein
Source: eLife. 2024 May 24;12:RP90762. doi: 10.7554/eLife.90762 (PMC11126310; doi:10.7554/eLife.90762)
Supplement: Figure 1—source data 1. [file elife-90762-fig1-data1.pdf]

Figure 1b-source data 1 Raw and annotated image for the PAGE gel stained in Coomassie Blue

Coomassie blue
Raw image

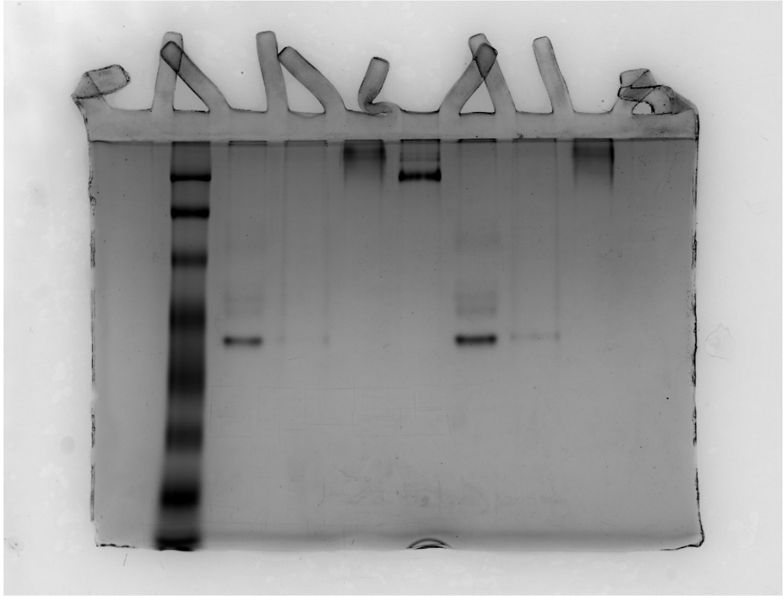

Coomassie blue
Annotated

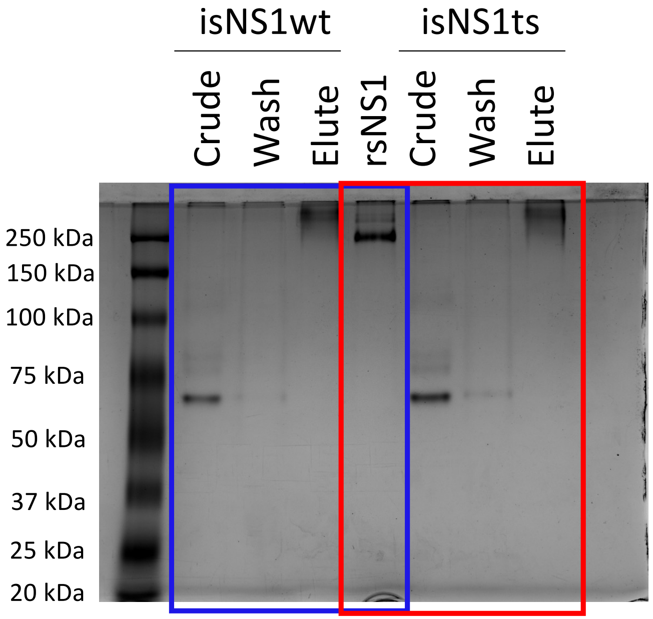

Remarks: Left side of rsNS1 blots are in the main figures as it is the WT NS1 (in blue), while the right side of rsNS1 blots are in the supp which is the mutant NS1 data (in red)
