# Supplementary material for: Secreted dengue virus NS1 from infection is predominantly dimeric and in complex with high-density lipoprotein
Source: eLife. 2024 May 24;12:RP90762. doi: 10.7554/eLife.90762 (PMC11126310; doi:10.7554/eLife.90762)
Supplement: Figure 1—source data 2. [file elife-90762-fig1-data2.pdf]

Figure 1b-source data 2 Raw and annotated image for the western blot analysis (anti-NS1)

Western blot

Raw image

Longer exposure

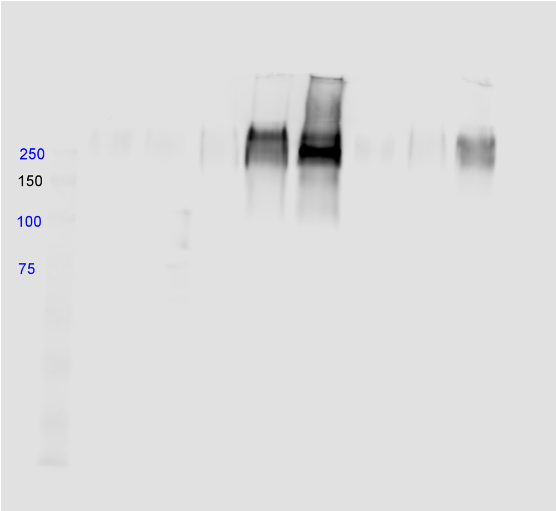

Shorter exposure

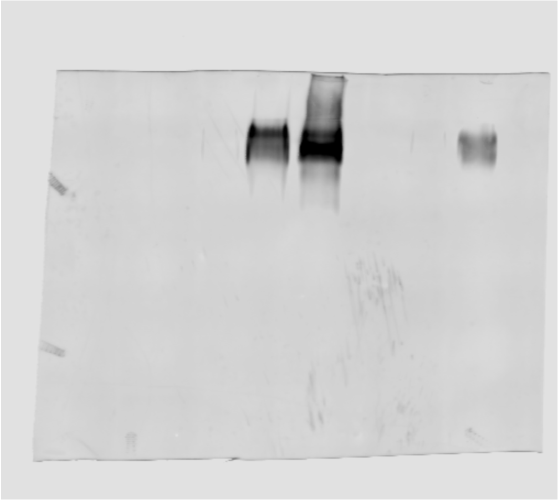

Western blot

Annotated

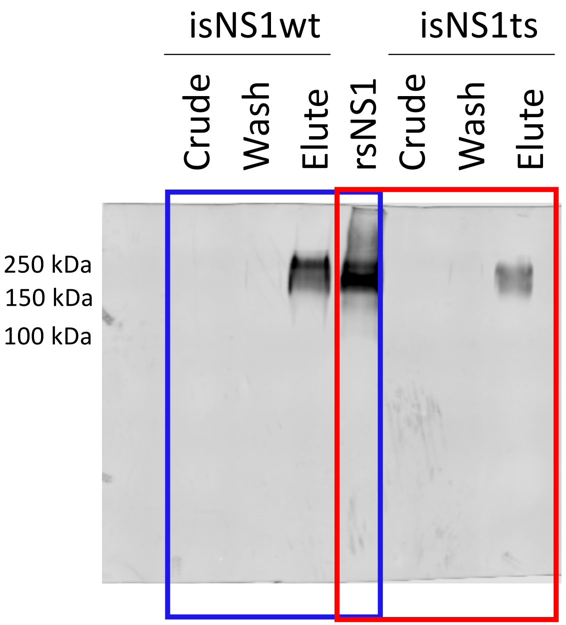

Remarks: Fig. 1b uses the western blot image with a shorter exposure as the bands are more clearly defined but the ladder is masked. Hence, the western blot image with a longer exposure is shown side by side to indicate the ladder markings, annotated directly after the blot was imaged. In the annotated version (of the blot with a shorter exposure), isNS1wt samples (crude, wash and elute) are shown to the left of rsNS1 (Fig. 1c) (in blue), while isNS1ts samples (crude, wash and elute) are shown to the right of rsNS1 (Supp Fig. 3a) (in red).
