# Supplementary material for: Secreted dengue virus NS1 from infection is predominantly dimeric and in complex with high-density lipoprotein
Source: eLife. 2024 May 24;12:RP90762. doi: 10.7554/eLife.90762 (PMC11126310; doi:10.7554/eLife.90762)
Supplement: Figure 1—source data 3. [file elife-90762-fig1-data3.pdf]

Figure 1c-source data 1 Raw and annotated image for the PAGE gel stained in Coomassie Blue

Coomassie blue

Raw image

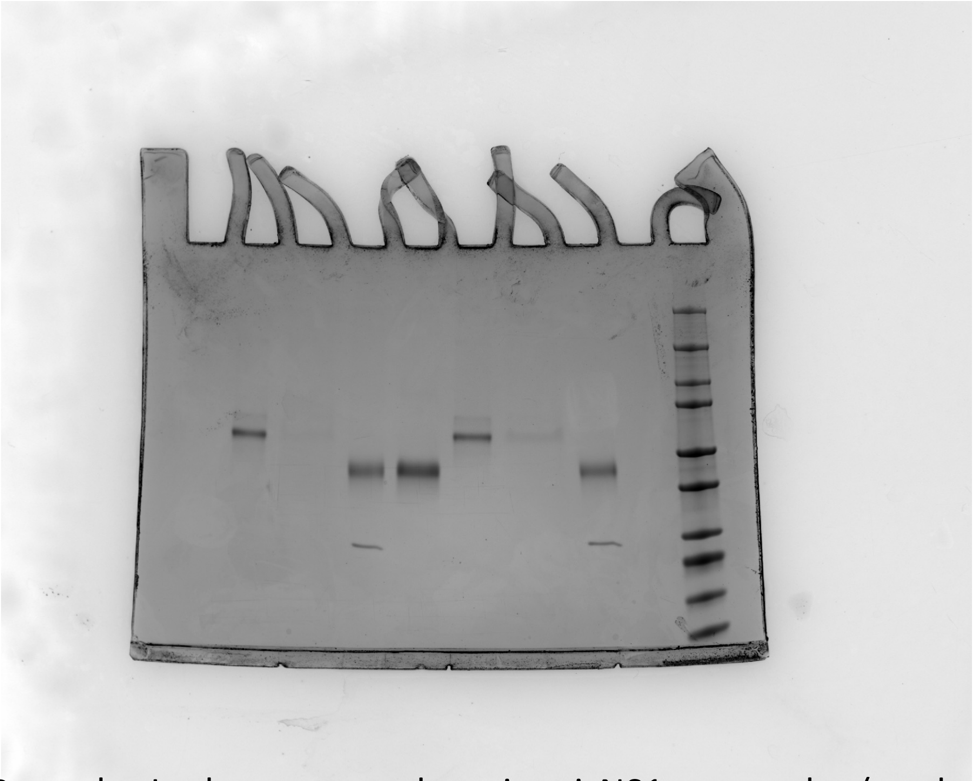

Coomassie blue

Annotated

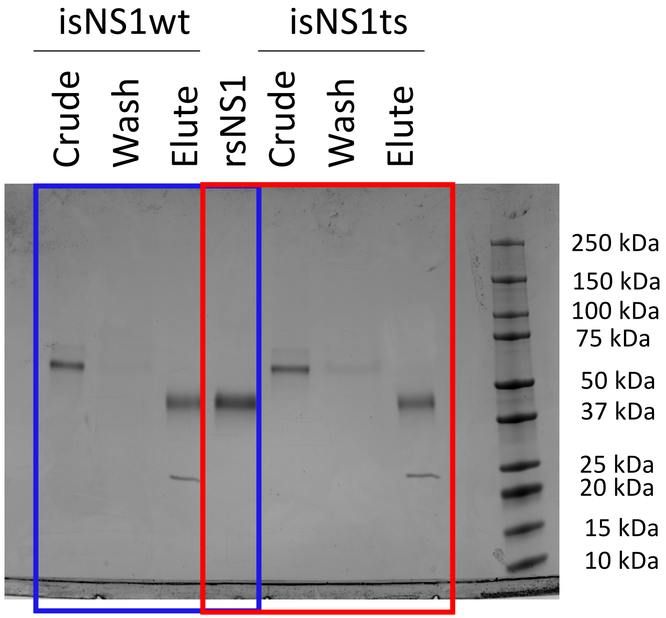

Remarks: In the annotated version, isNS1wt samples (crude, wash and elute) are shown to the left of rsNS1 (Fig. 1c) (in blue), while isNS1ts samples (crude, wash and elute) are shown to the right of rsNS1 (Supp Fig. 3b) (in red).
