# Supplementary material for: Secreted dengue virus NS1 from infection is predominantly dimeric and in complex with high-density lipoprotein
Source: eLife. 2024 May 24;12:RP90762. doi: 10.7554/eLife.90762 (PMC11126310; doi:10.7554/eLife.90762)
Supplement: Figure 1—source data 4. [file elife-90762-fig1-data4.pdf]

Figure 1c-source data 2 Raw and annotated image for the western blot analysis (anti-NS1)

Western blot (NS1)

Raw image

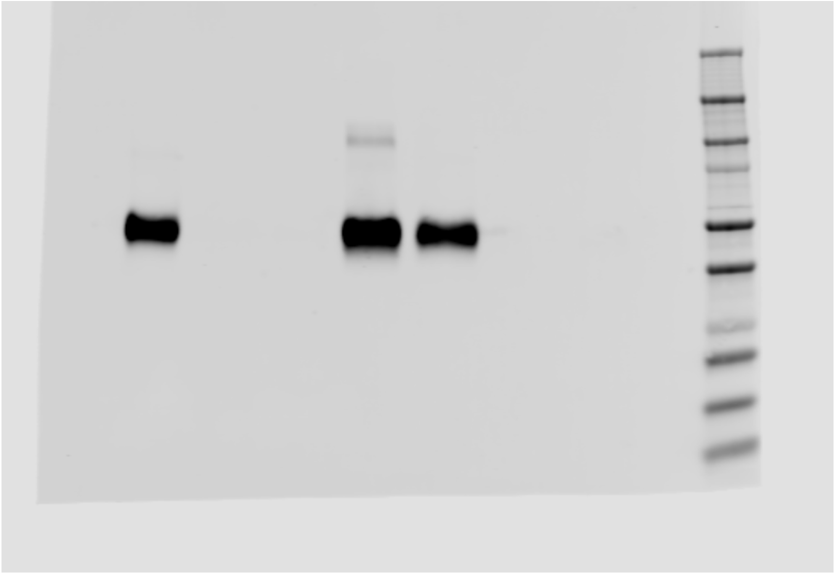

Western blot (NS1)

Annotated

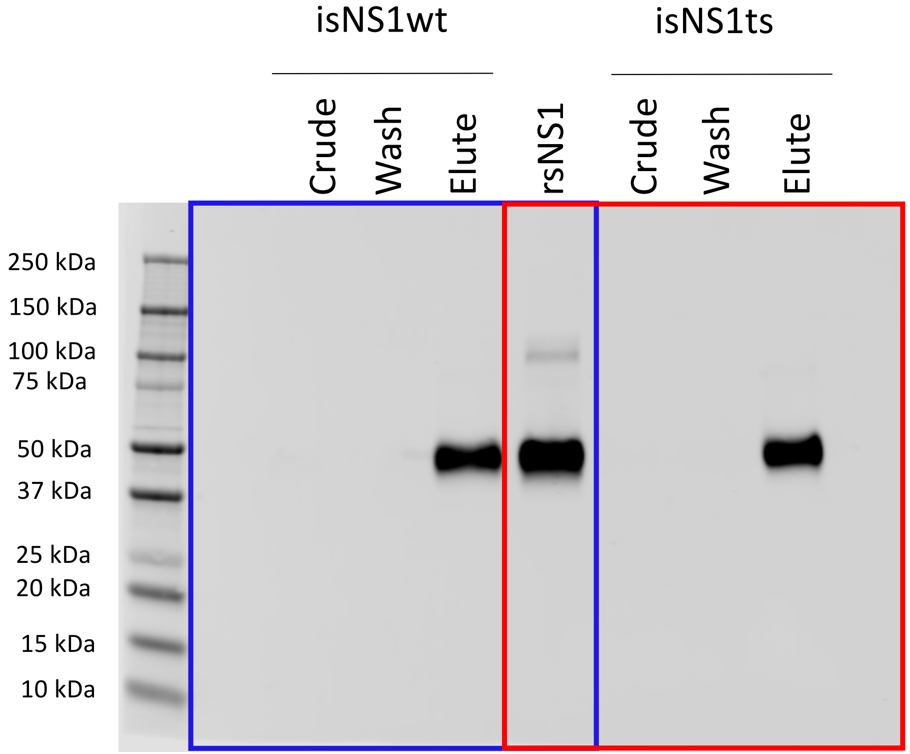

Remarks: Raw image is flipped horizontally. Annotated version on the right shows the correct orientation, where isNS1wt samples (crude, wash and elute) are shown to the left of rsNS1 (Fig. 1c) (in blue), while isNS1ts samples (crude, wash and elute) are shown to the right of rsNS1 (Supp Fig. 3b) (in red).
