# Supplementary material for: Secreted dengue virus NS1 from infection is predominantly dimeric and in complex with high-density lipoprotein
Source: eLife. 2024 May 24;12:RP90762. doi: 10.7554/eLife.90762 (PMC11126310; doi:10.7554/eLife.90762)
Supplement: Figure 1—figure supplement 2—source data 2. [file elife-90762-fig1-figsupp2-data2.pdf]

Figure 1-figure supplement 2-source data 2 Raw and annotated image for the PAGE gel stained in Coomassie Blue for isNS1ts

Coomassie Blue

Raw image

Native-PAGE

Reducing SDS-PAGE

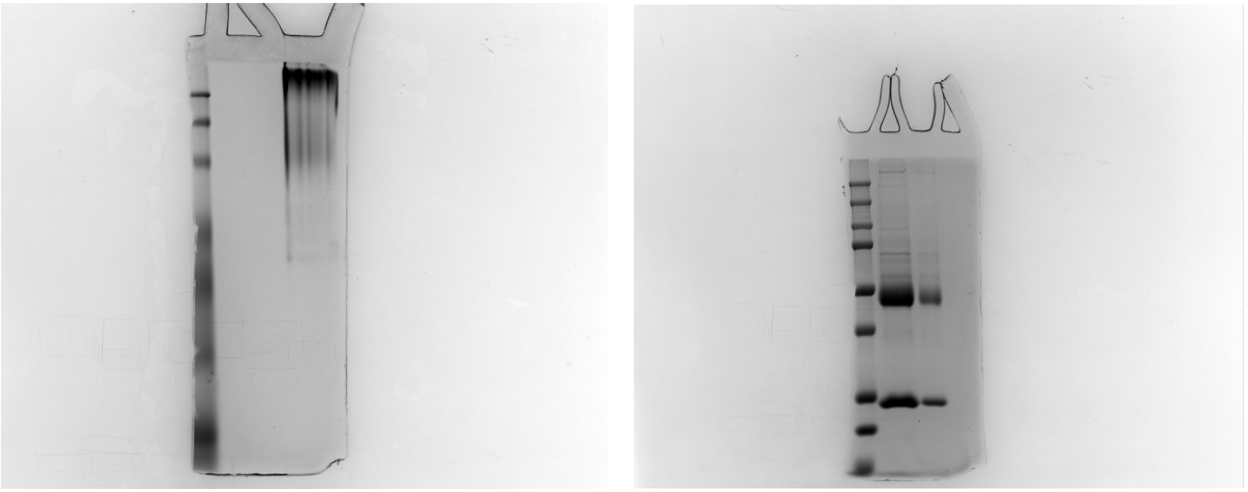

Coomassie Blue

Annotated

Native-PAGE

Reducing SDS-PAGE

isNS1ts

isNS1ts

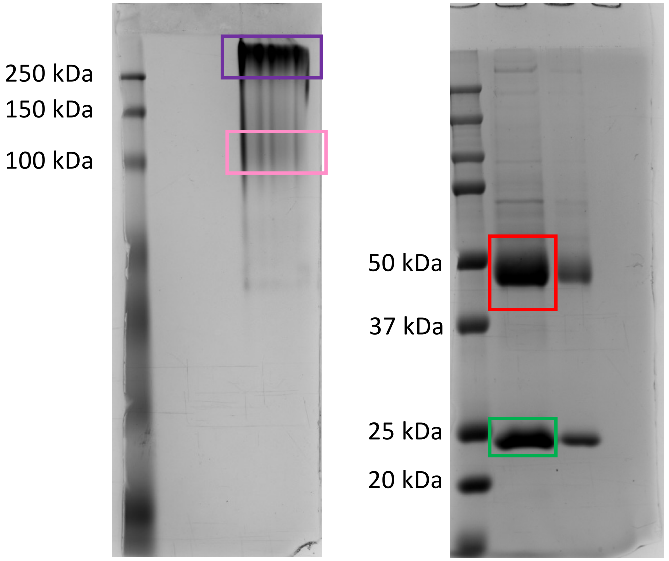

Remarks: Source data of reducing SDS-PAGE cropped gel image shown in the manuscript (red and green box) is shown in source data 3 instead
