# Supplementary material for: Secreted dengue virus NS1 from infection is predominantly dimeric and in complex with high-density lipoprotein
Source: eLife. 2024 May 24;12:RP90762. doi: 10.7554/eLife.90762 (PMC11126310; doi:10.7554/eLife.90762)
Supplement: Figure 1—figure supplement 2—source data 3. [file elife-90762-fig1-figsupp2-data3.pdf]

Figure 1-figure supplement 2-source data 3 Raw and annotated image for the SDS-PAGE gel stained in Coomassie Blue for isNS1wt and isNS1ts presented in Figure 1-figure supplement 2b

Coomassie Blue

Raw image

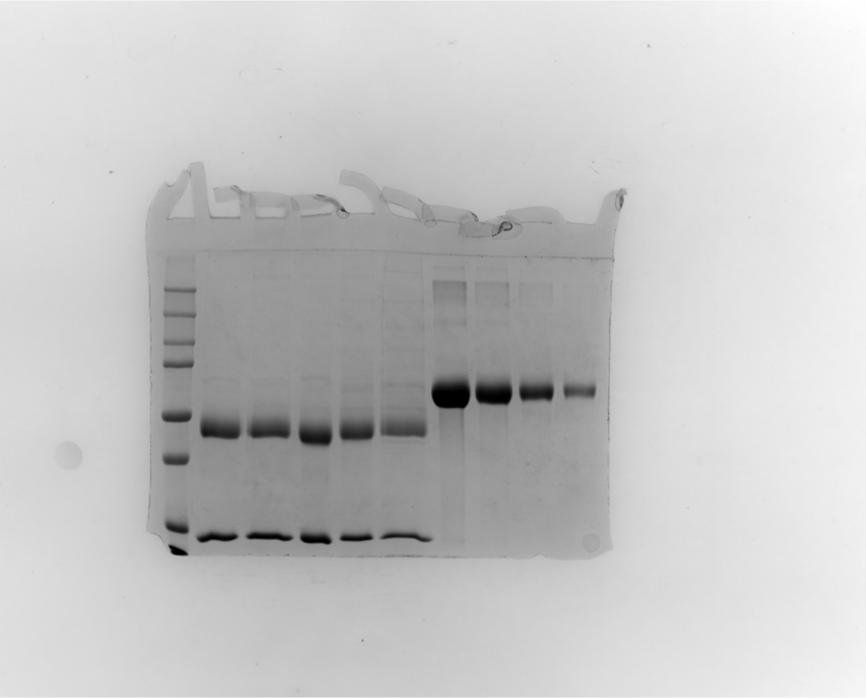

Coomassie Blue

Annotated

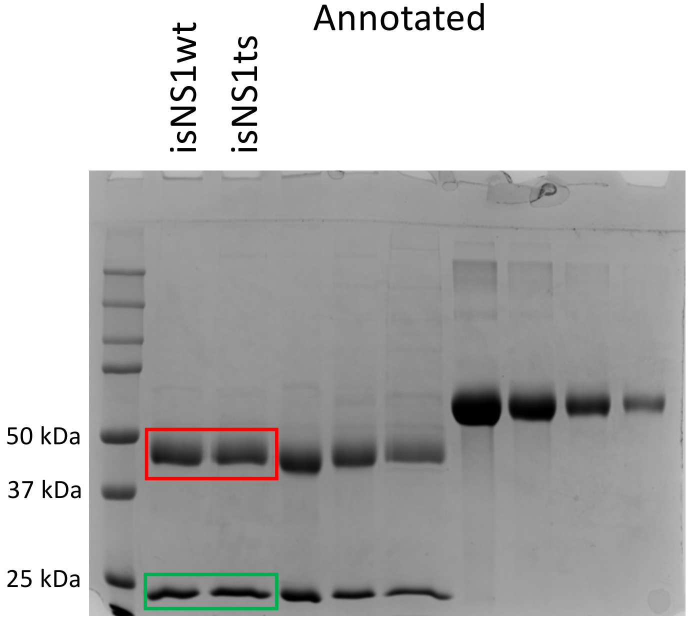

Remarks: Boxed up in red and green is the cropped gel image shown in the manuscript, this is representative of the same results shown in source data 1-2.
