# Supplementary material for: Secreted dengue virus NS1 from infection is predominantly dimeric and in complex with high-density lipoprotein
Source: eLife. 2024 May 24;12:RP90762. doi: 10.7554/eLife.90762 (PMC11126310; doi:10.7554/eLife.90762)
Supplement: Figure 4—source data 1. [file elife-90762-fig4-data1.pdf]

Figure 4-source data 1 Raw and annotated image for the PAGE gel visualized using silver stain

Silver Stain

Raw image

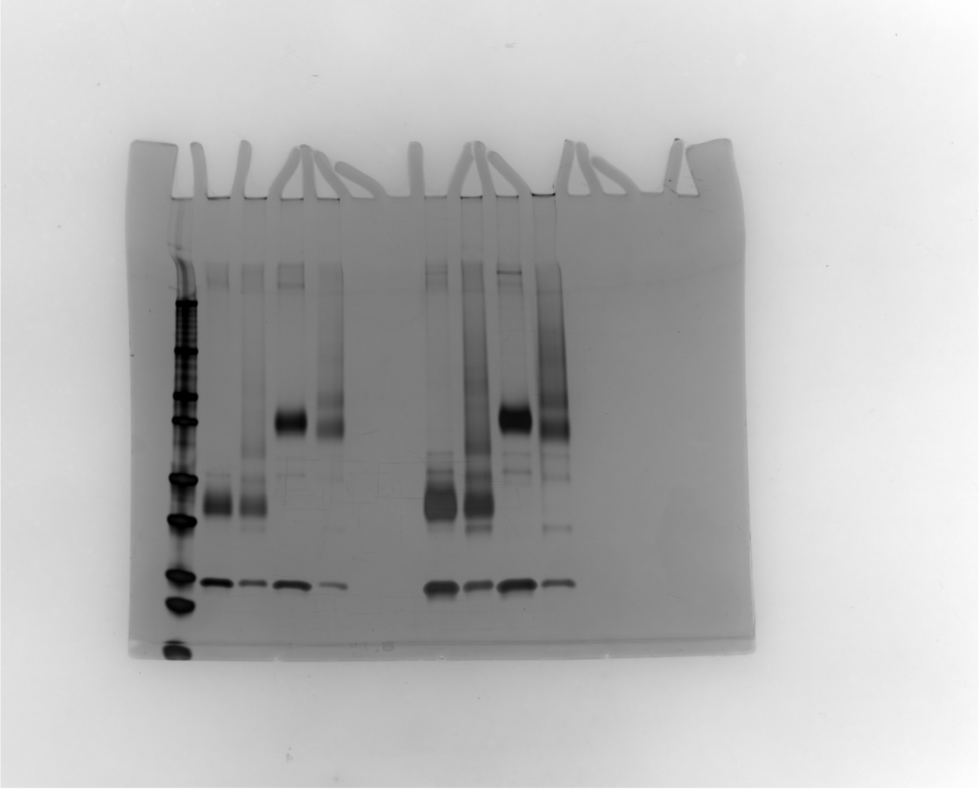

Silver Stain

Annotated

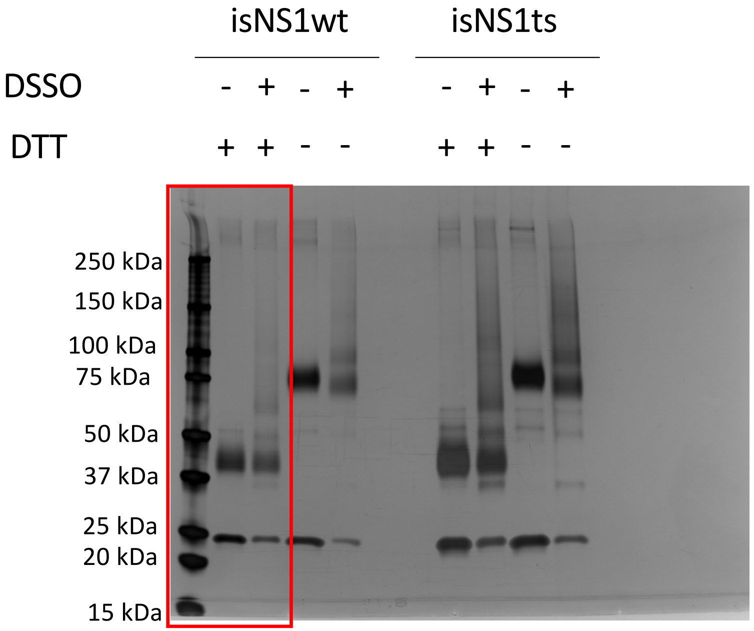

Remarks: Boxed up in red is the cropped gel image shown in the manuscript. The silver stain raw and annotated image is the same for Figure 4-figure supplement 1a for isNS1wt
