# Supplementary material for: Secreted dengue virus NS1 from infection is predominantly dimeric and in complex with high-density lipoprotein
Source: eLife. 2024 May 24;12:RP90762. doi: 10.7554/eLife.90762 (PMC11126310; doi:10.7554/eLife.90762)
Supplement: Figure 4—figure supplement 1—source data 1. [file elife-90762-fig4-figsupp1-data1.pdf]

Figure 4-figure supplement 1-source data 1 Raw and annotated image for the western blot analysis (anti-NS1) on an SDS-PAGE gel

Western blot (NS1)

Raw image

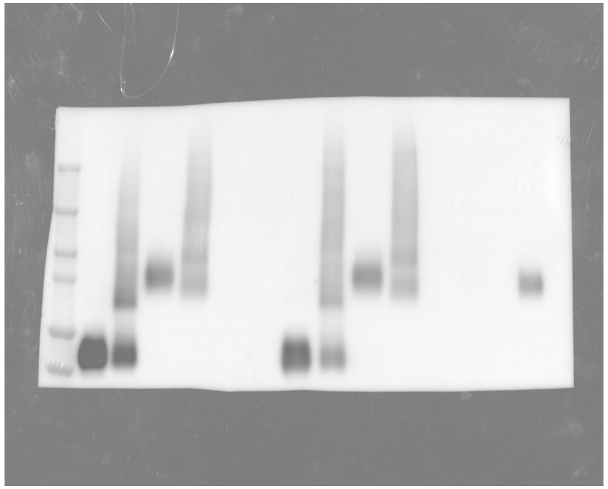

Western blot (NS1)

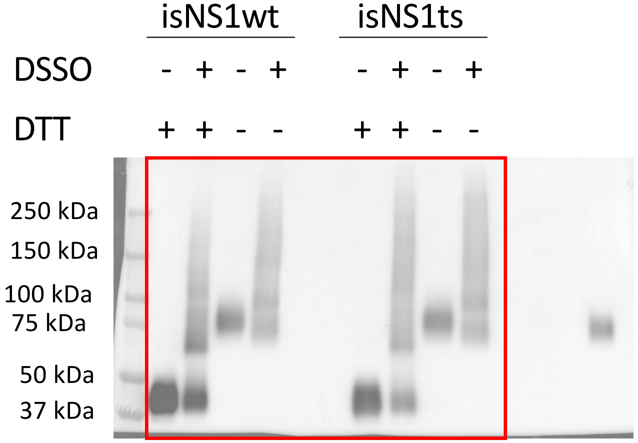

Remarks: Boxed up in red is the cropped WB image shown in the manuscript. The silver stain raw and annotated image is shown in source data for Figure 4a.
