# Supplementary material for: Secreted dengue virus NS1 from infection is predominantly dimeric and in complex with high-density lipoprotein
Source: eLife. 2024 May 24;12:RP90762. doi: 10.7554/eLife.90762 (PMC11126310; doi:10.7554/eLife.90762)
Supplement: Figure 4—figure supplement 1—source data 2. [file elife-90762-fig4-figsupp1-data2.pdf]

**Figure 4-figure supplement 1-source data 2 Raw and annotated image for the western blot analysis (anti-NS1) on a Native gel**

silver stain

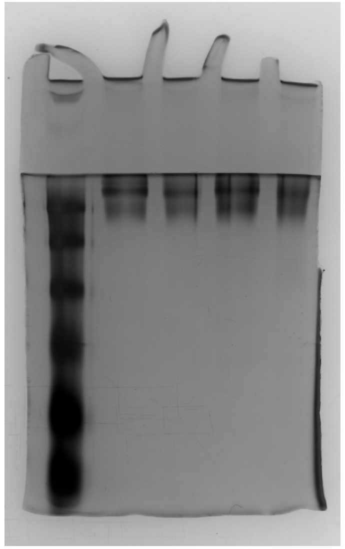

Western blot (NS1)

Raw image

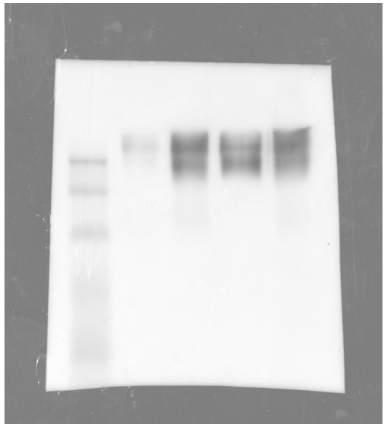

Western blot (NS1)

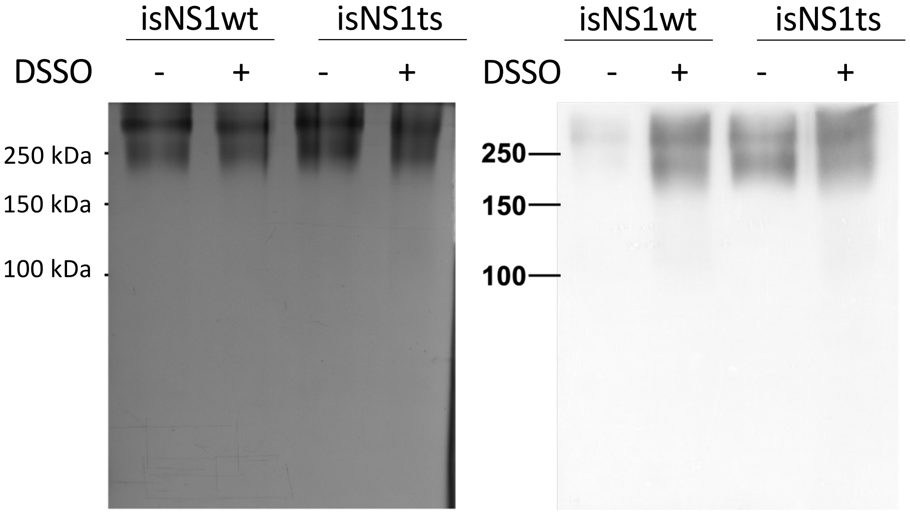


Remarks: -
